# Toxicological Stability of *Ocimum basilicum* Essential Oil and Its Major Components in the Control of *Sitophilus zeamais*

**DOI:** 10.3390/molecules26216483

**Published:** 2021-10-27

**Authors:** Eridiane da Silva Moura, Lêda Rita D’Antonino Faroni, Fernanda Fernandes Heleno, Alessandra Aparecida Zinato Rodrigues

**Affiliations:** 1 Department of Agricultural Engineering, Universidade Federal de Viçosa, Viçosa 36570-900, MG, Brazil; annne.moura@gmail.com (E.d.S.M.); fernandafhy@yahoo.com.br (F.F.H.); alessandra.rodrigues@ufv.br (A.A.Z.R.); 2 Serviço Autônomo de Água e Esgoto, Senador Firmino 36540-000, MG, Brazil; 3 Department of Chemistry, Universidade Federal de Viçosa, Viçosa 36570-900, MG, Brazil

**Keywords:** storage, monoterpenes, bioinsecticide, insect pest, toxicity

## Abstract

Essential oils (EOs) are widely recognized as efficient and safe alternatives for controlling pest insects in foods. However, there is a lack of studies evaluating the toxicological stability of botanical insecticides in stored grains in order to establish criteria of use and ensure your efficiency. The objective of this work was to evaluate the toxicological stability of basil essential oil (*O. basilicum*) and its linalool and estragole components for *Sitophilus zeamais* (Motschulsky) adults in corn grains by fumigation. The identification of the chemical compounds of the essential oil was performed with a gas chromatograph coupled to a mass selective detector. Mortality of insects was assessed after 24 h exposure. After storage for six (EO) and two months (linalool and estragole) under different conditions of temperature (5, 20, and 35 °C) and light (with and without exposure to light), its toxicological stability was evaluated. Studies revealed that the essential oil of *O. basilicum* and its main components exhibited insecticidal potential against adults of *S. zeamais*. For greater toxicological stability, suitable storage conditions for them include absence of light and temperatures equal to or less than 20 °C.

## 1. Introduction

Essential oils (EOs) are classified as secondary metabolites produced by various parts of the plant such as seeds, stems, leaves, and flowers. They are mixtures of volatile, natural substances, characterized by strong odor and, in most cases, have lipophilic constitution [1]. As they are composed of volatile terpenoids such as monoterpenes (C_10_) and sesquiterpenes (C_15_) and phenylpropenes (derived from the phenyl group junction (aromatic ring) and a three-carbon side chain (propyl group) [2], which usually originate from various biosynthesis pathways [3], there are a wide variety of possible applications of essential oils [4]. Among the current applications of EOs is their use as an alternative to synthetic insecticides, as EOs have great biocidal potential, presenting insect toxicity [5].

EOs and their compounds are believed to have a higher barrier to pest resistance and lower risk to human health and environmental contamination compared to conventional insecticides [6]. Among the essential oils with insecticidal activity is the essential oil of *Ocimum basilicum*, aromatic and medicinal plant of the Lamiaceae family [7], composed mainly of linalool and estragole [8,9].

The toxicity of *O. basilcium* essential oil has already been proven for *Acanthoscelides obtectus* (Coleoptera: Chrysomelidae) [10], *Rhyzopertha dominica* (Coleoptera: Bostrichidae) [11], *Sitophilus zeamais* (Coleoptera: Curculionidae) [12], *Tribolium castaneum* (Coleoptera: Tenebrionidae) [13], *Zabrotes subfasciatus* (Coleoptera: Chrysomelidae) [14], *Anopheles funestus* (Diptera: Culicidae) [15] and *Sitophilus Oryzae* (Coleoptera: Curculionidae) [16]. Because this essential oil is mainly composed of linalool and estragole, its toxicity to stored grain pest insects can be explained by the action of mixed inhibition of the enzyme acetylcholinesterase (AChE) caused by such compounds, especially when they are applied by fumigation [17,18].

Linalool, the most well-known monoterpene of *O. basilicum* essential oil, is present in the essential oil of various medicinal plants, mainly of the Lamiaceae family [19]. It is insect repellent [20], inhibits the reproduction of *Acanthoscelides obtectus* (Say) (Coleoptera: Chrysomelidae) [21] and exhibits larvicidal activity against *Culex quinquefasciatus* and *Aedes stephensi* larvae (Diptera: Culicidae) [22,23].

Estragole, a volatile monoterpenoid ether found in numerous plants [24]. This component and its biotransformation products have toxic potential because they are genotoxic, mutagenic or carcinogenic [25]. Nevertheless, it was considered safe (GRAS—Generally Recognized As Safe) by FEMA (Flavor and Extract Manufacturer’s Association, 2008) as it does not pose a risk to human health in small quantities (0.6 mg kg^−1^ day^−1^). Estragole inhibits the growth of *Aedes aegipyti* (Diptera: Culicidae) larvae and has antiparasitic and antihelmintic actions [26]. This compound has reported potential insecticide for *Oryzaephilus surinamensis* (Coleoptera: Silvanidae), *Lasioderma serricorne* (Coleoptera: Anobiidae), *Liposcelis bostrychophila* (Psocoptera: Liposcelididae) and *Tribolium castaneum* (Coleoptera: Tenebrionidae) [27,28,29,30,31].

Given that *O. basilicum* EO and its major components linalool and estragole are toxic to stored grain pest insects and knowing that once deprived of the protective compartmentalization of the plant, essential oil constituents are especially prone to oxidative damage, chemical transformations, or polymerization through enzymatic or chemically triggered reactions by external factors such as temperature and light [32]. the objective of this work was to determine the toxicological stability of *O. basilicum* EO and its linalool and estragole components on fumigation *Sitophilus zeamais* in corn grains, after storage as a function of temperature and luminosity for a period of six (EO) and two months (linalool and estragol).

## 2. Material and Methods

### 2.1. Insect Colony

The insects were raised on maize grains with water content of 12.1% (wet basis) under constant conditions of temperature (25 ± 2 °C), relative humidity (70 ± 5%) and scotophase 24 h. For the creation were used 3 L glass vials, closed with perforated plastic lid and internally coated with organza to allow gas exchange.

### 2.2. Essential Oil

The essential oil used in the research was acquired through the company Mundo dos Óleos (Brasília, DF, Brazil). 100% pure and natural oil extracted from *O. basilicum* leaves by steam distillation, obtained from selected raw material, to preserve the main properties of each extracted element, as well as enhance its flavor, color, and aroma characteristics. All the essential oil used in the research was acquired on the same date, thus belonging to the same manufacturing batch, in order to avoid interference in the research due to compositional variability.

### 2.3. Essential Oil Analysis

The analysis of the chemical composition of the essential oil was performed at the Department of Chemistry of the Federal University of Viçosa in Viçosa, Minas Gerais, Brazil. *O. basilicum* essential oil was analyzed by mass spectrometry coupled gas chromatography (GC-MS) on a QP2010 model equipment (Shimadzu, Tokyo, Japan) under the following conditions: fused silica capillary column (30 m in length) and 0.25 mm internal diameter) with RTX^®^-5MS stationary phase (0.25 µm film thickness) and helium as a carrier gas with a flow rate of 1.0 mL/min. Injector temperature of 220 °C, the initial column temperature was 60 °C, with programming to increase by 2 °C until reaching a temperature of 200 °C, and 5 °C until reaching a maximum temperature of 250 °C. Mass spectra were obtained by electron impact at 70 eV, with 29 to 400 (*m*/*z*) scan. 1 µL of the prepared oil solution was injected at a concentration of 10 mg mL^−1^ with a split ratio of 1:20. The main constituents were identified and quantified by their retention index (IR) relative to the hydrocarbon standard (C_7_–C_30_) (99%, Supelco, Bellefonte, PA, USA) and confirmed by comparing the mass spectrum of the compounds with the NIST 14 spectrotheque. 

### 2.4. Exposure to Temperature and Light Radiation

For the evaluation of the effect of temperature on the stability of the essential oil and its major compounds, clear glass containers wrapped in foil and properly sealed, 20 mL of *O. basilicum* essential oil and 1 mL of each compound were under different temperature conditions. The flasks were divided into three lots and packaged for six months for EO and two months for linalool and estragole, in the following environments: refrigerator at 5.0 ± 1 °C (low temperature); in an incubator chamber (model 347, CD, Fanem, São Paulo, SP, Brazil) at a temperature of 20 ± 2 °C (average temperature) and an incubator chamber at a temperature of 35 ± 2 °C (high temperature).

For the evaluation of light stability, clear glass vials containing the essential oil and its linalool and estragole compounds were kept in a B.O.D. (model 347 CD, Fanem, São Paulo, SP, Brazil) at a temperature of 20 ± 2 °C and subjected to light from cold white lamps (100 W each) (Philips, São Paulo, SP, Brazil) for six months for EO and two months for linalool and estragole.

### 2.5. Toxicological Stability

The fumigation bioassays were performed in 0.8 L (8 cm diameter × 15 cm high) glass vials with 50 non-sexed *S. zeamais* adults, in four replications. The concentrations of *O. basilicum* essential oil stored under different conditions ranged from 8 to 40 μL L^−1^ of air. Working solutions of the essential oil were prepared with toluene solvent (Sigma-Aldrich, 99.9%, Baden-Württemberg, Germany) and applied with a microsyringe (Hamilton, Reno, NV, USA) on 4 mm diameter paper filter discs. 4 cm placed in Petri dishes (6.5 cm diameter). Petri dishes were covered with organza type tissue and placed at the base of the flasks. Pure solvent (toluene) was used as a control. The vials were sealed with a screw-on metal cap and sealed with parafilm (PM996, American, NV, USA) after insect distribution to prevent oil vapor leakage during the exposure period. The flasks were kept in an incubator chamber at a temperature of 27 ± 2 °C for 24 h. After this period, dead and living insects were counted. Corrected mortality was calculated by Abbott’s formula [33].

Pure linalool and estragole were purchased from Sigma-Aldrich (Burlington, MA, USA). Toxicity assays were performed at concentrations ranging from 8 to 40 μL L^−1^. Each filter paper disc (4.4 cm) was treated with 25 μL of toluene diluted linalool and estragole solution and placed in a Petri dish (6.5 cm in diameter), covered with organza and inserted into the base of glass pots with a capacity of 0.8 L. A total of 50 non-sexed adults were placed by pot to expose the insects to the fumigant activity of the compounds for 24 h. Each treatment consisted of four repetitions. As a control 25 μL of pure toluene was used.

### 2.6. Statistical Analysis

Toxicity data were subjected to probit analysis using SAS software (SAS Institute, Cary, NC, USA), generating concentration-mortality curves. Mortality data were submitted to ANOVA and Tukey test with Statistica 8 software (StatSoft Inc., Tulsa, OK, USA).

## 3. Results and Discussion

### 3.1. Essential Oil Composition

**Figure 1:**
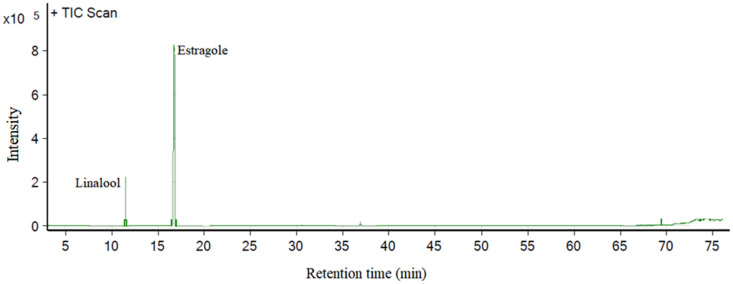
Chromatogram of *Ocimum basilicum* essential oil (10 mg mL^−1^ in toluene).

The relative chemical composition of the essential oil compounds of *O. basilicum* leaves were performed by GC-MS. The major constituents were identified by their retention index (RI) relative to a homologous series of n-alkanes and confirmed by comparing the mass spectrum of the compounds with the NIST 14 spectrotheque. Chromatographic analysis showed that estragole (H_2_C=CHCH_2_C_6_H_4_OCH_3_) and linalool ((CH_3_) 2C=CHCH_2_CH_2_C (CH_3_)(OH)CH=CH_2_) were the major components of *O. basilicum* essential oil (Figure 1), representing 85% and 12% of the identified compounds, respectively.

These compounds are responsible for most of the composition of this essential oil [8,9]. Generally, linalool and estragole are the major components of *O. basilicum* EO, but factors such as soil type, altitude, temperature, insolation period, cultivation, drying conditions and storage influence its composition [31], explaining variations in the amount of the compounds.

### 3.2. Toxicological Stability of Essential Oil

The Probit model was adequate for the concentration-mortality data of all fumigation treatments, based on the low χ^2^ value and the high *p* value obtained from the *O. basilicum* essential oil curves stored under different conditions and their components. linalool and estragole over *S. zeamais*. For the untreated essential oil, the values of χ^2^ = 0.14 and *p* = 0.98 were obtained. Lethal concentrations to cause 50 and 95% insect mortality (LC_50_ and LC_95_) were 25.4 µL L^−1^ and 178.4 µL L^−1^ of air, respectively (Table 1). The slope of the curve was (1.94 ± 0.37), which indicates genetic homogeneity among individuals of the *S. zeamais* population.

*O. basilicum* EO was lethal to *S. zeamais* by fumigation [34], but the effectiveness of essential oils depends on factors such as dose or concentration, insect species, application surface, penetration pathway, method of application and composition of oil, season, ecological conditions, method and extraction time, plant part and storage conditions [35,36]. Both the untreated EO and the EO stored under different temperatures (5 and 20 °C) and exposed to light for a period of six months had lower LC_50_ when applied by fumigation.

The LC_50_ value of untreated *O. basilicum* essential oil when applied by fumigation (25.4 µL L^−1^ air) was lower than that of *Minthostachys verticillata* (28.2 µL L^−1^ air) and *Eucalyptus globulus* essential oil (335.7 µL L^−1^ of air) [37] and higher than *Melaleuca alternifolia* essential oil (7.7 µL L^−1^ of air) for *S. zeamais* [38]. The fumigant activity of *O. basilicum* EO on *S. zeamais* can be explained by the fact that monoterpenoids inhibit the acetylcholinestrase (AChE) nerve conduction enzyme [37]. In addition, studies have shown that essential oils can significantly inhibit the activity of two detoxifying enzymes in *S. zeamais*, glutathione S-transferase (GST) and carboxylesterase (CarE), as well as negatively regulating differentially expressed genes (DEGs) in response to fumigation [37].

**Figure 2:**
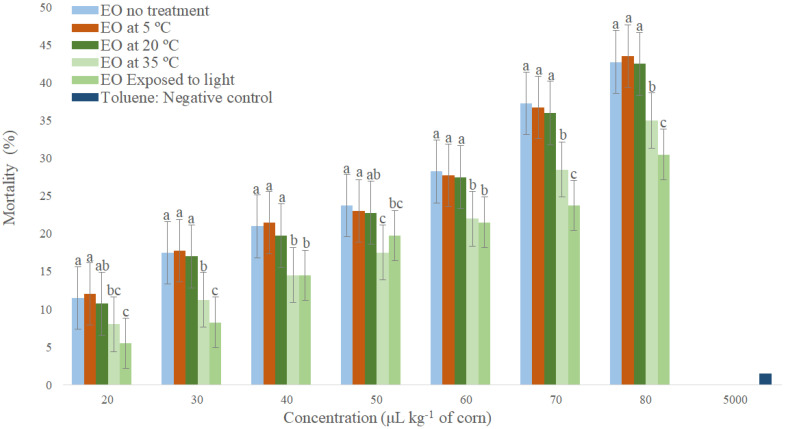
Toxicological stability by fumigation of *Ocimum basilicum* essential oil stored under different conditions and their major components for *Sitophilus zeamais*. Means followed by the same letter in the column do not differ at 5% probability by Tukey test.

*O. basilicum* EO caused higher mortality of *S. zeamais* when compared to the negative control, showing that it has higher fumigant activity (Figure 2). The *O. basilicum* EO stored at 5 to 20 °C and without storage, differed statistically from each other in only three concentrations (8; 16 and 40 µL L^−1^ of air), indicating that temperatures up to 20 °C do not interfere significantly on the toxicological stability of EO for *S. zeamais* adults when stored for six months. When comparing the EO without storage and EO stored at 35 °C and in light exposure, there was a statistical difference in all concentrations (Figure 2). The EO stored at 35 °C was more stable than the EO stored in light exposure, causing higher mortality of *S. zeamais* adults, which shows that light exposure decreases the toxicity of *O. basilicum* EO on *S. zeamais*.

**Figure 3:**
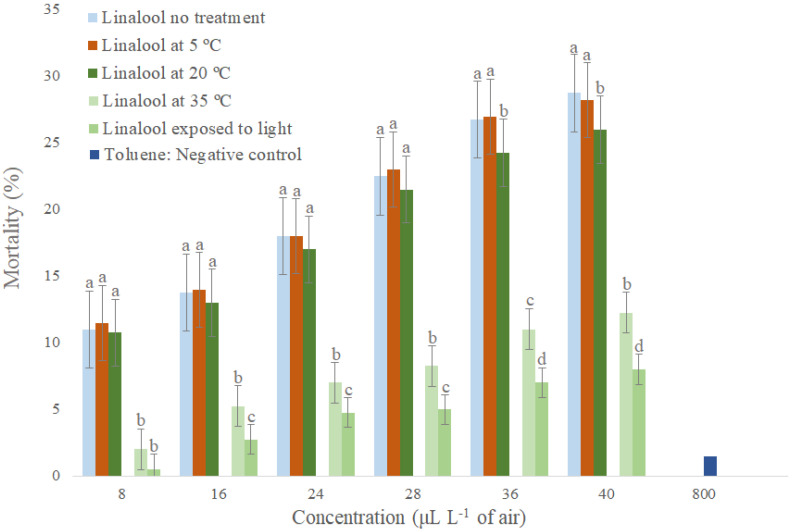
Toxicological stability by fumigation of linalool stored under different conditions for *Sitophilus zeamais*. Means followed by the same letter in the column do not differ at 5% probability by Tukey test.

When comparing the untreated EO and the EO stored at 35 °C in the fumigation applications, there was a statistical difference in all concentrations (Figure 2 and Figure 3). This indicates that storage at high temperatures for a period of six months affects its toxicological stability on *S. zeamais* adults. The temperature plays a crucial role in the degradation process of essential oils, which directly affects their stability. This decisively influences the stability of the essential oil in several respects [38]. Generally, chemical reactions accelerate with increasing heat due to temperature dependence of the reaction rate, as expressed by the Arrhenius equation [39]. Based on this, Van’t Hoff’s law states that a temperature increase of 10 °C doubles chemical reaction rates, a ratio that can be consulted to predict stability at different temperatures [40].

Increasing temperature advances the self-oxidation and decomposition processes of hydroperoxides, as heat can contribute to free radical formation [41]. Essential oils vary in their susceptibility to self-oxidation at different storage temperatures. In general, monitoring of volatile plant extracts and essential oil composition demonstrates that stability decreases with prolonged storage time, as well as a temperature rise from 0 to 28 °C [42], 4 to 25 °C [43] and 23 to 38 °C [44].

There was a statistical difference between the untreated EO and the EO stored in light exposure at all concentrations (Figure 2 and Figure 3). This indicates that six-month storage in light exposure affects its toxicological stability in *S. zeamais* adults. This is possibly due to the presence of ultraviolet light (UV) and visible light (Vis) being responsible for accelerating the self-oxidation processes in the essential oils, triggering what results in free radical formation [44]. Auto-oxidation involves a succession of chemical reactions that alter the initial composition of the oil, leading to the production of low molecular weight compounds and oxidized polymers, as well as the destruction of important fatty acids and the formation of other compounds, compromising their stability [42].

Comparison of EO stored at 35 °C and in light exposure shows that the toxicological stability of *O. basilicum* EO over *S. zeamais* was most affected by storage in light exposure. The light is much more important than temperature in the oxidation of essential oils [42], although the effect of light on oil oxidation is lessened with increasing temperature [45]. The effect of sunlight for 2 h caused degradation of the quality of ginger oil, while it remained stable when stored in the dark for the same period of time [46].

Processing and storage of oils in exposure to light can lead to the generation of a wide range of undesirable compounds, some of which are harmful to health because of their high toxicity, thereby altering their stability [47]. Among the components of essential oils, monoterpenes have been shown to degrade rapidly under the influence of visible light [48]. The same study also showed that there were transformation reactions in marjoram oil during storage under visible light, which led to the formation of several unidentified elements and smaller components.

### 3.3. Toxicological Stability of Linalool and Estragole

The Probit model was adequate for concentration-mortality data, based on the low χ^2^ values and the high *p* values obtained on the linalool and estragole curve stored under different conditions over *S. zeamais*. For untreated linalool, the values of χ^2^ = 5.57 and *p* = 0.34 were obtained. Lethal concentrations to cause 50% and 95% insect mortality (LC_50_ and LC_95_) were 34.6 µL L^−1^ and 330.3 µL L^−1^ of air, respectively (Table 2). The slope of the curve was (1.67 ± 0.22), which indicates genetic homogeneity among individuals of the *S. zeamais* population.

Linalool caused higher mortality of *S. zeamais* adults when compared to the negative control, showing that it has higher fumigant activity (Figure 3). Studies have shown that essential oil components are able to inhibit cellular respiration enzymes, nervous system enzymes such as acetylcholinesterase (AChE), and detoxification system enzymes such as P450 and esterase [49], which weakens the insecticide metabolism in insects. Linalol acts together with other compounds in the cholinergic system of insects, promoting the rapid breakdown of the nervous system [50]. Linalool stored at 5 to 20 °C and without storage differed statistically from each other in only two concentrations (36 and 40 µL L^−1^ of air), indicating that temperatures up to 20 °C do not significantly affect its toxicological stability for adults of *S. zeamais* during storage for two months. When comparing non-stored linalool and linalool stored at 35 °C and in light exposure, there was a statistical difference in all concentrations except one (8 µL L^−1^ of air) (Figure 3). Linalool stored at 35 °C was more stable than linalool stored in light exposure, causing higher mortality of *S. zeamais* adults, which shows that light exposure is more detrimental to linalool stability than temperature increase.

For untreated estragole, the values of χ^2^ = 5.48 and *p* = 0.35 were obtained. Lethal concentrations to cause 50% and 95% insect mortality (LC_50_ and LC_95_) were 38.13 µL L^−1^ and 314.01 µL L^−1^ of air, respectively (Table 3). The slope of the curve was (1.79 ± 0.22), which indicates genetic homogeneity among individuals of the *S. zeamais* population.

**Figure 4:**
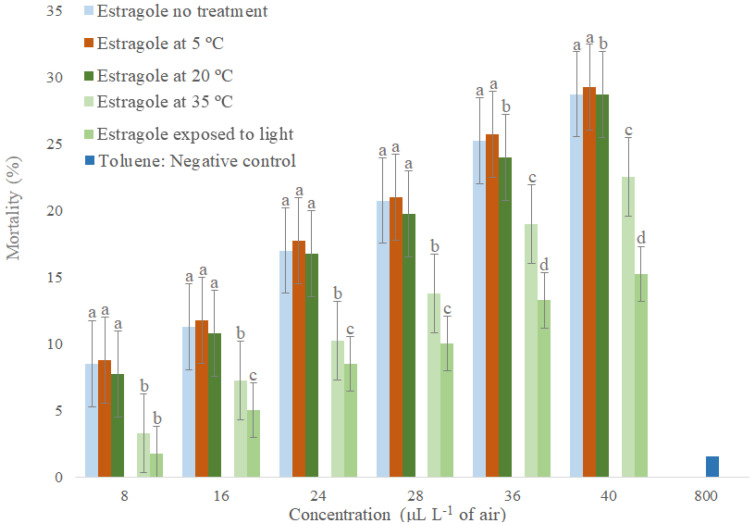
Toxicological stability by estragole fumigation stored under different conditions for *Sitophilus zeamais*. Means followed by the same letter in the column do not differ at 5% probability by Tukey test.

Estragole caused higher mortality of *S. zeamais* adults when compared to the negative control, showing that it has higher fumigant activity (Figure 4). Estragole stored at 5 to 20 °C and without storage differed statistically from only one concentration (32 µL L^−1^ of air), indicating that temperatures up to 20 °C do not significantly affect its toxicological stability for adults of *S. zeamais* during storage for two months. Comparing estragole without storage and linalool stored at 35 °C and in light exposure, there was a statistical difference in all concentrations, indicating that the increase of storage temperature decreases the toxicity of estragol for adults of *S. zeamais* (Figure 4). Estragole stored at 35 °C differed statistically from estragole stored in light exposure by only one concentration (16 µL L^−1^ of air), which shows that both treatments decrease the toxicity of estragole for *S. zeamais* adults to the same extent.

Linalool LC_50_ and LC_95_ increased from 34.56 to 132.4 µL L^−1^ of air and from 330.3 to 495.6 µL L^−1^ of air respectively when stored at 35 °C for two months. The same occurred with estragol LC_50_ and LC_95_ which increased from 38.1 to 56.5 µL L^−1^ of air and from 314.0 to 395.2 µL L^−1^ of air, respectively. This shows that storage at 35 °C decreases the toxicity of this compound for *S. zeamais* adults. This can be explained by the fact that temperature directly influences the stability of volatile compounds [41]. Generally, chemical reactions accelerate with increasing heat due to temperature dependence of the reaction rate, as expressed by the Arrhenius equation [42]. Terpenoids, especially terpenes and aldehydes, are known to be susceptible to rearrangement processes at elevated temperatures. Terpenic conversion reactions by heating have been reported for both isolated compounds [51,52] and for essential oils [53]. Mircene, for example, suffered degradation as it was exposed to higher storage temperatures for 120 days. The initial percentage of mircene fell from 17.38% (6 °C) to 3.99% at 37.5 °C [54].

In this work, the comparison between linalool stored at 35 °C and stored at 20 °C in light exposure shows that it was more toxicologically stable on *S. zeamais* when exposed to 35 °C, indicating that light exposure is more detrimental to the stability of linalool than increase in temperature. This is because some monoterpenes are more thermodynamically stable while others demonstrate rapid degradation under the influence of visible light [50]. Linalol, for example, is 5.9 kJ mol ^−1^ more stable than geraniol [55].

In contrast to linalool, the toxicological stability of estragole on *S. zeamais* was more affected by increased storage temperature (35 °C) than by exposure to light. This can occur due to the evaporation process of low boiling compounds, mainly hydrocarbons and sesquiterpenes [56].

There was no significant difference between linalol and estragole stored at 5 and 20 °C, which shows that they can be stored in this temperature range for two months without decreasing their toxicological stability on *S. zeamais*. Three temperatures (4 °C in a cold room, −20 °C in a freezer and 25 °C at room temperature) were used to assess the stability of *Thymus daenensis* essential oil for three months [57]. The results indicated that at room temperature, the amounts of thymol and carvacrol increased considerably by 26.6% and 23% after 3 months, respectively. The increase in thymol and carvacrol by storage at room temperature represents an increase in oil quality index. In addition, oil compositions exhibited the smallest changes and maintained primary quality when stored at low temperatures, particularly at 20 °C [57].

## 4. Conclusions

The essential oil of *O. basilicum* and its linalool and estragole components components exhibited insecticidal potential against *S. zeamais* adults in corn grains by fumigation. Increasing temperature (35 °C) and exposure to light during storage negatively affects the stability of *O. basilicum* EO, reducing its toxicity against *S. zeamais*. Aiming at the higher toxicity of *O. basilicum* EO to *S. zeamais*, the storage conditions suitable for it are at temperatures of maximum 20 °C and without exposure to light.

## Figures and Tables

**Table 1:** Lethal concentrations of *Ocimum basilicum* essential oil stored under different conditions and their major components for fumigation *Sitophilus zeamais*.

Components	LC_50_ (FI 95%) (µL L^−1^ of Air)	LC_95_ (FI 95%) (µL L^−1^ of Air)	Inclination (±MSE ^1^)	χ^2^ (d*f*)	*p*
Essential oil	25.4 (23.1–28.6)	178.4 (161.2–196.2)	1.94 ± 0.37	0.14 (7)	0.98
EO at 5 °C	25.7 (23.7–29.1)	172.3 (154.3–187.2)	1.99 ± 0.37	0.09 (7)	0.99
EO at 20 °C	25.8 (24.1–30.6)	151.3 (139.4–192.3)	2.13 ± 0.38	0.85 (7)	0.83

LC = Lethal Concentration (µL L^−1^ of air); FI = Fiducial Interval; MSE ^1^ = Mean square error; χ^2^ = Chi square; *p* = Probability; df = degrees of freedom; EO = Essential oil.

**Table 2:** Lethal concentrations of linalool stored under different conditions for fumigation *Sitophilus zeamais*.

Components	LC_50_ (FI 95%) (µL L^−1^ of Air)	LC_95_ (FI 95%) (µL L^−1^ of Air)	Inclination (±MSE ^1^)	χ^2^ (d*f*)	*p*
Linalool	34.6 (31.6–41.2)	330.3 (269.8–463.6)	1.67 ± 0.22	5.57 (5)	0.34
Linalool at 5 °C	33.9 (30.7–42.1)	339.3 (301.3–498.9)	1.64 ± 0.22	6.07 (5)	0.29
Linalool at 20 °C	39.4 (33.5–48.3)	382.7 (365.2–529.8)	1.51 ± 0.22	6.91 (5)	0.22
Linalool at 35 °C	132.4 (110.3–198.7)	495.6 (427.5–612.6)	1.39 ± 0.19	1.56 (5)	0.98
Linalool exposed to light	178.9 (152.4–215.6)	534.2 (518.3–725.5)	1.53 ± 0.23	1.84 (5)	0.96

LC = Lethal Concentration (µL L^−1^ of air); FI = Fiducial Interval; MSE ^1^ = Mean square error; χ^2^ = Chi square; *p* = Probability; df = degrees of freedom.

**Table 3:** Lethal concentrations of estragole stored under different conditions for *Sitophilus zeamais* by fumigationis.

Components	LC_50_ (FI 95%) (µL L^−1^ of Air)	LC_95_ (FI 95%) (µL L^−1^ of Air)	Inclination (±MSE ^1^)	χ^2^ (d*f*)	*p*
Estragole	38.1 (35.6–45.7)	314.0 (298.3–465.6)	1.79 ± 0.22	5.48 (5)	0.35
Estragole at 5 °C	37.2 (34.3–43.8)	305.4 (287.1–419.4)	1.79 ± 0.22	6.08 (5)	0.29
Estragole at 20 °C	41.0 (38.9–49.2)	335.0 (303.5–490.2)	1.80 ± 0.23	4.38 (5)	0.49
Estragole at 35 °C	56.5 (47.5–63.4)	395.2 (347.6–511.5)	1.94 ± 0.25	3.44 (5)	0.63
Estragole exposed to light	53.6 (45.9–61.3)	362.1 (323.7–501.7)	1.98 ± 0.17	6.96 (5)	0.43

LC = Lethal Concentration (µL L^−1^ of air); FI = Fiducial Interval; MSE ^1^ = Mean square error; χ^2^ = Chi square; *p* = Probability; df = degrees of freedom.

## Sample Availability

Samples of the compounds are not available from the authors.
